# Immunohistochemical Characterization of Leukocytic Subpopulations in Chronic Endometritis

**DOI:** 10.1155/S1064744996000555

**Published:** 1996

**Authors:** Ossama Tawfik, Susan Venuti, Sharla Brown, Julie Collins

**Affiliations:** 1 Department of Pathology and Laboratory Medicine University of Kansas Medical Center 3901 Rainbow Boulevard Kansas KS 66160 USA; 2 Department of Obstetrics and Gynecology University of Kansas Medical Center Kansas KS USA

## Abstract

*Objective:* We analyzed the histologic and immunohistochemical changes in the endometrial leukocytic subpopulations to determine which of them are characteristic of chronic endometritis.

*Results:* Endometrial biopsies from 25 cases of chronic endometritis and 35 controls were studied. Characteristic morphologic findings included the presence of a plasma cell infiltrate, and a prominent, albeit non-specific, lymphocytic infiltrate in all patients with endometritis. A neutrophilic infiltrate was also noted in 90% of the patients. Other non-specific histologic findings included occasional prominent lymphoid aggregates, stromal edema, hemorrhage, and necrosis and cystic dilation of some glands in endometria of patients with chronic endometritis. Endometrial immune cells were investigated immunohistochemically using antibodies to CD3 (pan-T), CD20 (pan-B, L26), and Ham-56 (macrophage). In control patients, endometrial immune cells were predominantly composed of CD3 and Ham-56 positive cells. CD20 positive cells comprised <2% of immune cells in control patients [mean: <2 cells/high power field (HPF)]. Large numbers of CD20 and CD3 lymphocytes were seen in endometria of patients with chronic endometritis. A semiquantitative analysis showed that the numbers of CD20 and CD3 positive cells in patients with chronic endometritis were increased 50- and 3-fold, respectively, when compared to those of control patients (mean B cells: 49 vs. 2 cells/HPF; mean T cells: 149 vs. 45 cells/HPF). CD20 positive cells were predominantly seen in subepithelial and periglandular aggregates. CD3 positive cells had a predominant stromal distribution and an occasional intra- or subepithelial localization. There was no difference in the number and distribution of Ham-56 positive cells in patients with or without endometritis.

*Conclusions:* These findings suggest that CD20 cells may have a significant role in the pathogenesis of chronic endometritis and that immunostaining for B and T lymphocytes could be used in confirming the diagnosis of endometritis or in diagnosing subclinical or progressing endometritis in which plasma cells could not be detected or are rarely identified.


Infectious Diseases in Obstetrics and Gynecology 4:287–293 (1996)
© 1997 Wiley-Liss, Inc.

### Immunohistochemical Characterization of Leukocytic Subpopulations in Chronic Endometritis

**Ossama Tawfik,[1]* Susan Venuti,[1] Sharla Brown,[2] and Julie Collins**
*[1]Department of Pathology and Laboratory Medicine, University of Kansas Medical Center, Kansas City, Kansas; [2]Department of Obstetrics and Gynecology, University of Kansas Medical Center, Kansas City, Kansas*

### Abstract

*Objective:* We analyzed the histologic and immunohistochemical changes in the endometrial leukocytic subpopulations to determine which of them are characteristic of chronic endometritis.

*Results:* Endometrial biopsies from 25 cases of chronic endometritis and 35 controls were studied. Characteristic morphologic findings included the presence of a plasma cell infiltrate, and a prominent, albeit non-specific, lymphocytic infiltrate in all patients with endometritis. A neutrophilic infiltrate was also noted in 90% of the patients. Other non-specific histologic findings included occasional prominent lymphoid aggregates, stromal edema, hemorrhage, and necrosis and cystic dilation of some glands in endometria of patients with chronic endometritis. Endometrial immune cells were investigated immunohistochemically using antibodies to CD3 (pan-T), CD20 (pan-B, L26), and Ham-56 (macrophage). In control patients, endometrial immune cells were predominantly composed of CD3 and Ham-56 positive cells. CD20 positive cells comprised <2% of immune cells in control patients [mean: <2 cells/high power field (HPF)]. Large numbers of CD20 and CD3 lymphocytes were seen in endometria of patients with chronic endometritis. A semiquantitative analysis showed that the numbers of CD20 and CD3 positive cells in patients with chronic endometritis were increased 50- and 3-fold, respectively, when compared to those of control patients (mean B cells: 49 vs. 2 cells/HPF; mean T cells: 149 vs. 45 cells/HPF). CD20 positive cells were predominantly seen in subepithelial and periglandular aggregates. CD3 positive cells had a predominant stromal distribution and an occasional intra- or subepithelial localization. There was no difference in the number and distribution of Ham-56 positive cells in patients with or without endometritis.

*Conclusions:* These findings suggest that CD20 cells may have a significant role in the pathogenesis of chronic endometritis and that immunostaining for B and T lymphocytes could be used in confirming the diagnosis of endometritis or in diagnosing subclinical or progressing endometritis in which plasma cells could not be detected or are rarely identified. Infect. Dis. Obstet. Gynecol. 4:287–293, 1996. © 1997 Wiley-Liss, Inc.

Key words
chronic endometritis; B lymphocytes; T lymphocytes; macrophages; neutrophils

Since first described by Hitschman and Adler, the presence of plasma cells in the endometrial stroma has been considered the hallmark for the histologic diagnosis of chronic endometritis.[1–10] Although characteristic nuclear and cytoplasmic features for plasma cells are well known, the search for them in endometrial stroma can be tedious in a large number of cases. Plasma cells may be obscured or mimicked by other cells. Macrophages and other stromal cells can occasionally be mis-

*Correspondence to: Dr. Ossama Tawfik, Department of Pathology and Laboratory Medicine, University of Kansas Medical Center, 3901 Rainbow Boulevard, Kansas City, KS 66160.

Received 6 September 1996
Accepted 15 November 1996

identified as plasma cells, particularly when they have an eccentrically located nucleus or a perinuclear halo. Chronic endometritis, occurring in the late secretory phase of the menstrual cycle, can occasionally be missed in a background of a pronounced predecidual reaction and an abundant lymphocytic infiltrate, which might hamper the search for plasma cells. In addition, the finding of occasional plasma cells in late menstrual and proliferative normal cyclic endometria casts some doubt in regard to the clinical significance of their presence.[6,7,9]

Attempts to correlate stromal acute and chronic inflammatory infiltrate, lymphoid follicles, stromal edema, and necrosis with chronic endometritis have been for the most part unfruitful.[4–6,8] It has generally been accepted that the presence of lymphocytes and/or neutrophils in the endometrium is neither specific nor important. The degree of chronic inflammation and lymphoid aggregates in particular is reported to be so common as to be considered normal findings in endometrial biopsies.[10,11]

In this study, we tested the hypothesis that the endometrium of women with chronic endometritis differs immunologically from that of patients with other conditions. Endometrial plasma cells, neutrophils, T and B lymphocytes, and macrophages were analyzed quantitatively and qualitatively in endometrial biopsies from patients with and without chronic endometritis.

### MATERIALS AND METHODS

### Tissues and Patient Demographics

Endometrial tissue from 60 patients treated at the University of Kansas Medical Center was used in this study. Twenty-five cases exhibited the characteristic histologic features of chronic endometritis, as described below. The mean age of these patients was 37.4 years (range 18–92 years). Eighteen patients were white, 4 African American, and 3 of other races. Clinically, 92% of cases (23/25) presented with irregular vaginal bleeding. One of the remaining 2 patients presented for fertility workup and the other for pelvic pain. The control group included 35 patients. Their mean age was 40.4 years (range 20–81 years). Of these patients, 25 were white, 9 African American, and 1 American Indian. Similar to the chronic endometritis group, the majority of the control group (71%, 25 cases) presented with irregular vaginal bleeding. Five patients had endometrial biopsies for fertility workup and one for amenorrhea. The clinical picture was not known for 4 patients.

### Immunoperoxidase Staining

Surgical specimens, fixed in 10% neutral buffered formalin and embedded in paraffin, were sectioned and stained with hematoxylin-eosin (HLE). Immunoperoxidase staining was performed using a labeled strepavidin-biotin peroxidase technique. Monoclonal antibodies used were directed against T lymphocytes (anti-CD3, 1:100), B lymphocytes (anti-CD20, L26, 1:25), and macrophages (Ham-56, 1:25) (Dako, Carpinteria, CA).

Briefly, the sections were deparaffinized in xylene and rehydrated in alcohol, followed by blockage of endogenous peroxide by incubation with 0.3% hydrogen peroxidase for 30 min. The primary antibody was applied to sections for 30 min, followed by incubation with biotinylated anti-immunoglobulin. Enzymatic treatment with 0.04% pepsin (10 min at 37°C) and 0.1% proteinase K (10 min at 37°C) was performed on the slides stained for CD3 and Ham-56, respectively. For color development, diaminobenzidine-hydrogen peroxide was used, creating a brown reaction product. Hematoxylin was used as a counterstain.

### RESULTS

### Morphologic Findings

Histologic findings in patients with chronic endometritis are summarized in Table 1. The endometrial glandular epithelium was proliferative in 4 patients, secretory in 5 patients, and indeterminate in 16 patients. A consistent feature was a prominent lymphoplasmacytic infiltrate in all cases of endometritis (Fig. 1A, B). The degree of plasma infiltrate varied considerably from one case to another. The mean plasma cell infiltrate was 26 cells/high power field (HPF) with a range of 2–95 cells/HPF. Plasma cells could be found randomly distributed throughout the endometrial stroma, but were mostly seen close to the surface epithelium and in close proximity to endometrial glands and blood vessels. None of the control endometria had plasma cell infiltrate. Lymphocytes were seen scattered throughout the endometrial stroma of all cases studied. Their distribution was neither specific nor different from that of control patients. Oc-

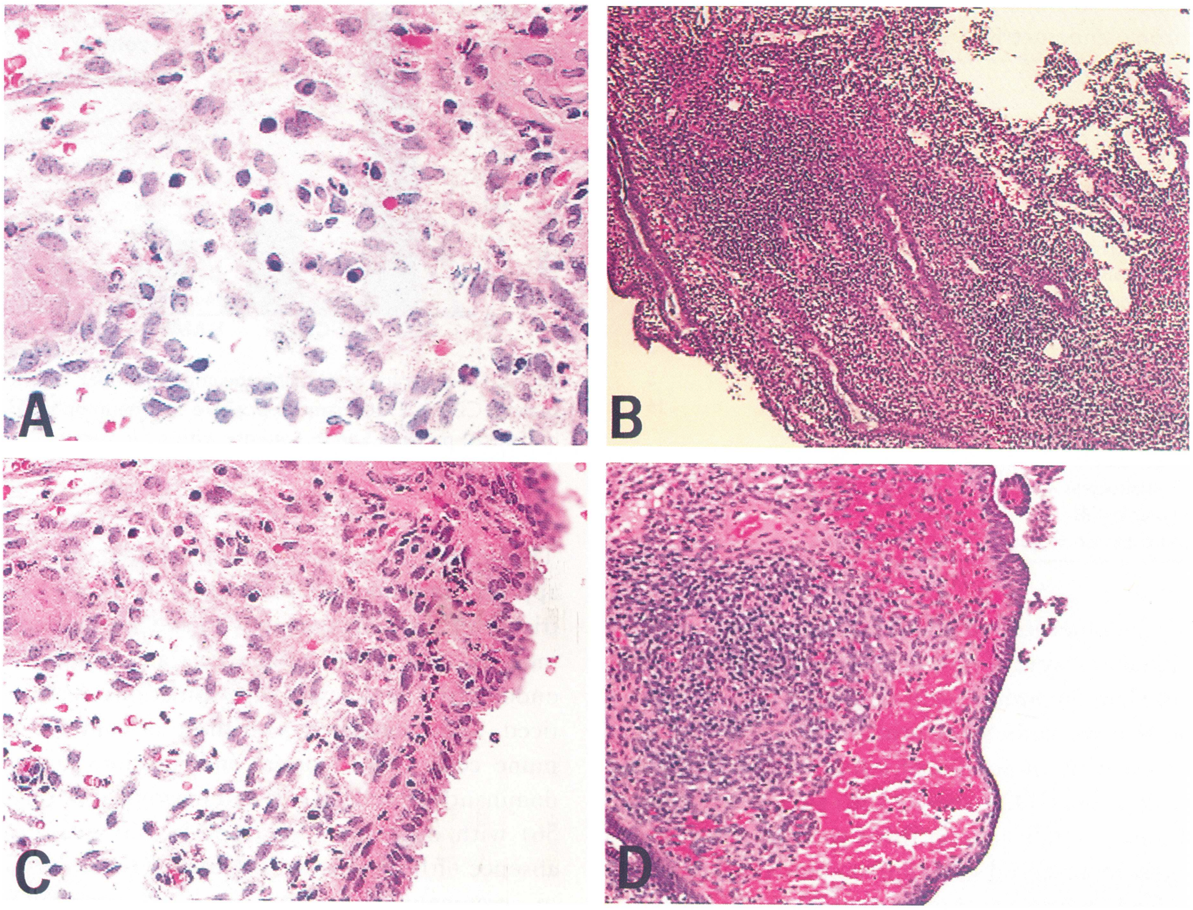

Fig. 1. Representative endometria of two patients with chronic endometritis demonstrating the characteristic histologic features with abundant plasma cell infiltrate in the stroma **(A)**, dense stromal chronic inflammation with prominent lymphoid aggregates **(B)**, submucosal acute inflammatory infiltrate **(C)**, and occasional stromal hemorrhage and edema **(D)**. H&E. ×40 (A); ×10 (B,D); ×20 (C).

casionally, prominent stromal lymphoid aggregates were noted in some patients with and without endometritis (Fig. 1B). Although lymphoid aggregates tended to be slightly larger in patients with endometritis, this finding was neither specific nor reliable. A stromal neutrophilic infiltrate was similarly found in 100% of patients with endometritis (Fig. 1C). The mean number of the endometrial neutrophils in patients with endometritis was higher than that of endometria from control patients [43 cells/HPF (range 7–98) vs. 13 cells HPF (range 1–80)]. Endometria in the secretory phase had slightly higher numbers of neutrophils compared to those in the proliferative phase [14 cells/HPF (range 1–74) vs. 9 cells/HPF (range 1–80)]. In contrast to lymphocytes, the neutrophilic infiltrate was only focal and sparse. It invariably involved the glandular and surface epithelium and the surrounding subepithelial stroma. Neutrophils were frequently seen within the surface epithelium forming microabscesses. Occasionally, a focal neutrophilic exudate was noted within the lumina.

The prevalence of edema, hemorrhage, and necrosis was also studied in the endometrial stroma of patients with endometritis. While superficial stromal edema was present in 90% of samples, stromal hemorrhage and necrosis were only noted in 40 and 7% of samples from patients with chronic endometritis, respectively (Fig. 1D, Table 1). Occasional cystic dilation of the glands was also seen in 10 cases (Table 1).

### Immunologic Findings

At the immunologic level, there was a significant difference in the number and distribution of endometrial leukocytes between patients with and with-

TABLE 1. Summary of the histologic features of chronic endometritis

| | No. of patients with changes in | |
|---|---|---|
| | Stroma | Glands |
| Stromal changes | | |
| Edema | 21 | — |
| Hemorrhage | 12 | — |
| Necrosis | 3 | — |
| Lymphoid aggregates | 9 | — |
| Phase | | |
| Proliferative | — | 4 |
| Secretory | — | 5 |
| Indeterminate | — | 16 |
| Leukocytic infiltrate | | |
| Plasma cells | 25 | — |
| Lymphocytes | 25 | — |
| Neutrophils | 25 | 21 |
| Cystic dilation of glands | — | 10 |

out endometritis. In control patients, endometrial leukocytes were almost entirely composed of CD3 and Ham-56 positive cells (Fig. 2). CD3 and Ham-56 positive cells were primarily localized in the stroma with an occasional intra- and subepithelial localization. CD20 positive cells were either totally absent or rarely seen in those patients and consistently represented <2% of endometrial leukocytes.

Endometria of chronic endometritis patients demonstrated a significant increase in the numbers of CD3 and CD20 positive cells (Fig. 2). A semi-quantitative analysis showed that the number of CD3 positive cells in the chronic endometritis patients was 3 times that of the control patients [149 cells/HPF (range 50–247) vs. 45 cells/HPF (range 8–129)]. The CD20 positive cells showed the most significant increase of 50 times, compared to those of controls [49 cells/HPF (range 7–112) vs. 2 cells/HPF (range 0–2)] (Fig. 2). The distribution of CD3 positive cells in chronic endometritis patients was similar to that of control patients, which was predominantly stromal (Fig. 3C,D). CD20 positive cells were mostly seen in subepithelial and periglandular aggregates (Fig. 3A,B). Occasional individual CD20 positive cells were also noted scattered throughout the stroma. A slight increase in the number of Ham-56 positive cells was seen in endometritis patients [38 cells/HPF (range 11–87) in patients with chronic endometritis vs. 31 cells/HPF (range 14–67) in controls]. However, their primary stromal distribution remained similar to that of control patients (Fig. 4).

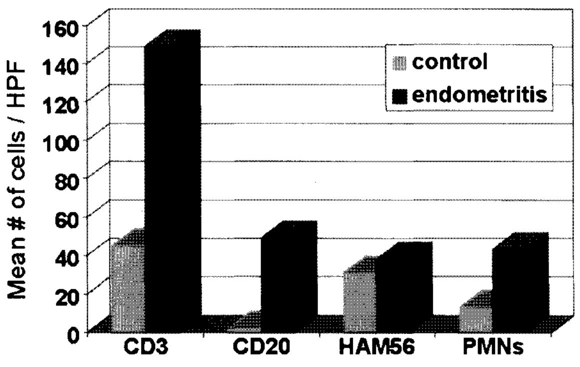

Fig. 2. Histogram comparing the mean number of endometrial CD3, CD20 Ham-56 positive and neutrophils/HPF in control patients and in patients with endometritis.

### DISCUSSION

The results of this study indicate that there are apparent immunologic differences at the endometrial level between women with and without endometritis. Changes in proportions and numbers of endometrial leukocyte subpopulations were noticed, when compared with those of controls. Immune cells in the normal endometrium are predominantly T cells (CD3) and macrophages (Ham-56) with occasional neutrophils and near total absence of B lymphocytes (CD20). Our results are in agreement with those previously reported, in which B lymphocytes comprised <2% of the endometrial leukocytes in control patients.[12–19] Variable numbers of neutrophils were scattered throughout. The immune cells were primarily localized in the endometrial stroma with rare intraepithelial and subepithelial accumulation of occasional T cells and macrophages. In patients with chronic endometritis, in addition to the presence of plasma cells, a significant finding was the marked increase in the number of B lymphocytes, which was consistently present in all patients. Furthermore, T cells and neutrophils showed a considerable increase in their numbers in endometritis patients, while macrophages were only slightly increased.

This study confirms the previously reported histologic features of chronic endometritis, including a prominent lymphoplasmacytic infiltrate in the endometrial stroma.[1–10] A focal but significant sub- and intraepithelial acute inflammatory infiltrate was also seen in the majority of cases with endometritis. Although lymphoid aggregates were repeatedly detected in most cases of endometritis, our results confirmed the previously reported data that there is

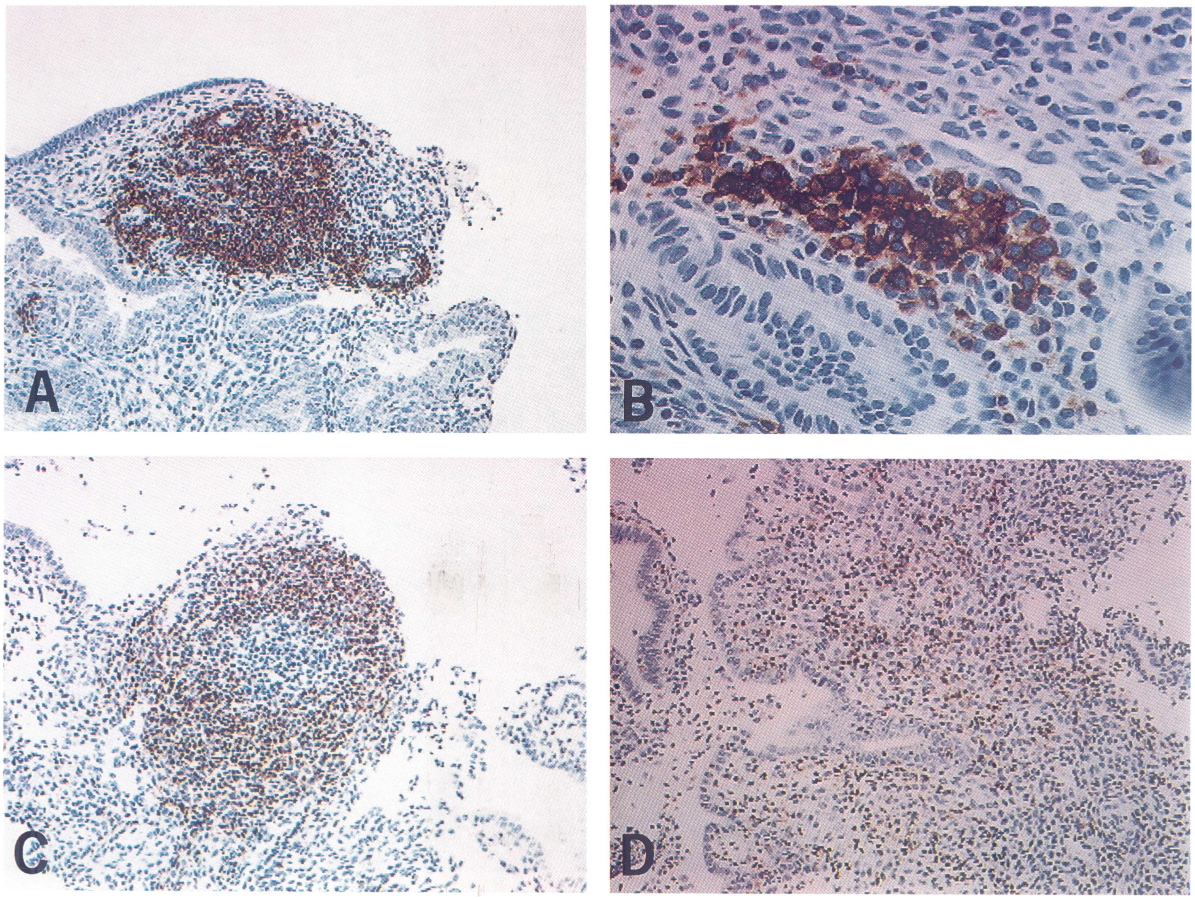

Fig. 3. **A,B:** Immunostaining for CD20 (L26, pan-B cell marker) positive cells in two representative patients with chronic endometritis. The stroma is focally infiltrated by positively stained CD20 positive cells that are predominantly arranged in aggregates. **C,D:** Immunostaining for CD3 (pan-T cell marker) positive cells in two representative patients with chronic endometritis. The CD3 positive cells are primarily localized in stroma in either aggregates or diffusely.

no significant correlation between lymphoid aggregates and the diagnosis of chronic endometritis.[10,11]

Traditionally, the presence of an unspecified number of plasma cells in the endometrial stroma is used by pathologists for the diagnosis of chronic endometritis.[1–8,10] In this study, the presence of at least 2 plasma cells was the minimum requirement for the diagnosis of endometritis. Several investigators have emphasized the shortcomings of such limited criteria and have urged further evaluation.[6,7,9] To the best of our knowledge, the relationship between B lymphocytes and endometritis has never been addressed. B cells are the precursors of the antibody-producing plasma cells. Two populations of lymphocytes are usually needed in an antibody response: helper T lymphocytes and B lymphocytes. The increase in B and T cell numbers under such circumstances is not surprising. Their proliferation may result from the antigenic stimulation exerted by the different organisms invading the endometrium and thus may represent the expression of a specific immune response directed against those organisms. An attempt to correlate the microbial type was not performed in this study. Kiviat et al.[9] have recently shown that the presence of low numbers of plasma cells in the endometrium had a low specificity for predicting the diagnosis of upper genital tract infection or laparoscopic salpingitis. The specificity and sensitivity of an accurate histologic diagnosis were significantly increased by the concurrent presence of 5 or more neutrophils within the endometrial surface and 1 or more plasma cells per × 120 field of tissue.[9]

The increased number of immune cells in chronic endometritis provides a host of clinically important implications. Immune cells can provide a

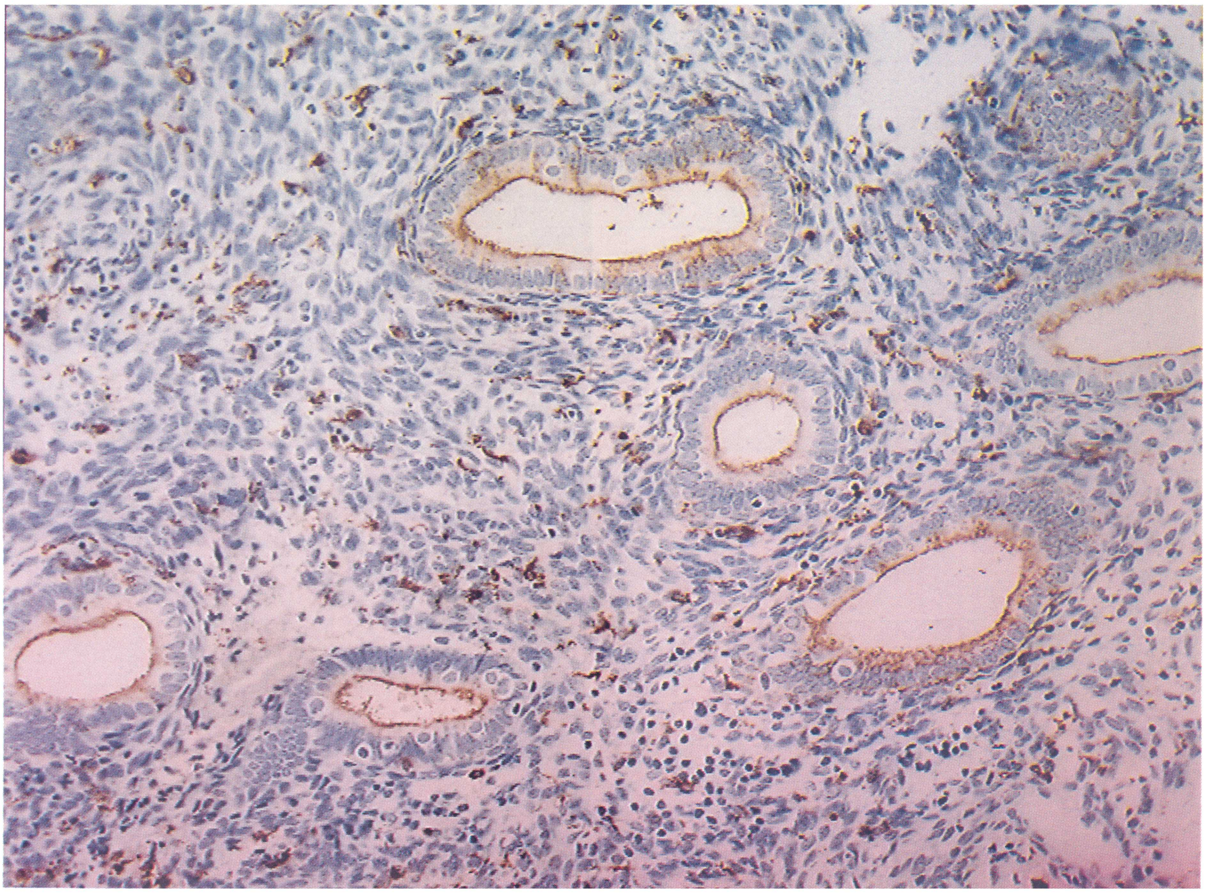

Fig. 4. Immunostaining for Ham-56 (macrophage marker) positive cells in a representative patient with chronic endometritis. Many stromal cells with the characteristic dendritic morphology of macrophages show strong cytoplasmic staining with Ham-56. The glandular epithelium showed a strong luminal staining pattern for Ham-56, as well.

mechanism for menstrual dysfunction, which is considered to be the most common presenting symptom in these patients. Furthermore, they have the ability to produce a variety of cytokines and growth factors that could have a harmful effect on pregnancy. Spontaneous abortion, infertility, and ectopic pregnancy are reported to be more common in these patients.[10,20–22]

Although the leukocyte quantitative method performed in this report is somewhat crude and time consuming, it has an advantage over automated image analysis techniques in which variations in staining intensity between sections and non-specific or background staining can affect results. In addition, biopsy specimens are usually not large enough and would not be sufficient for additional studies. The authors have taken extra care in the method of quantitation to reduce the potential for errors as much as possible.

The search for plasma cells remains the hallmark for the diagnosis of chronic endometritis. However, the data of this study suggest that immunohistochemical detection of other immune cells can provide important information not otherwise obtained by conventional histologic techniques. Although further confirmatory studies need to be performed, it is tempting to speculate that the identification of large numbers of B lymphocytes in the endometrium could be a useful and easy tool in diagnosing subclinical or progressing endometritis in which plasma cells cannot be detected or are rarely identified.

### REFERENCES

1. Dumoulin JG, Hughesdon PE: Chronic endometritis. J Obstet Gynaecol Br Emp 58:222–235, 1951.
2. Brudenell JM: Chronic endometritis and plasma cell infiltration of the endometrium. J Obstet Gynaecol Br Emp 62:269–274, 1955.

3. Jacobsen L, Westrom L: Objectivized diagnosis of acute pelvic inflammatory disease. Diagnostic and prognostic value of routine laparoscopy. Am J Obstet Gynecol 105: 1088–1098, 1969.
4. Cadena D, Cavanzo FJ, Leone CL, Taylor HB: Chronic endometritis. A comparative clinicopathologic study. Obstet Gynecol 41:733–738, 1973.
5. Rotterdam H: Chronic endometritis. A clinicopathologic study. Pathol Annu 13:209–231, 1978.
6. Greenwood SM, Moran TT: Chronic endometritis: Morphologic and clinical observations. Obstet Gynecol 58:176–184, 1981.
7. Burke RK, Hertig AT, Miele CA: Prognostic value of subacute focal inflammation of the endometrium with special reference to pelvic adhesions as observed on laparoscopic examination. J Reprod Med 30:646–650, 1985.
8. Winkler B, Gallo L, Reumann W, Richart RM, Mitao M, Crum CP: Chlamydial endometritis. A histological and immunohistochemical analysis. Am J Surg Pathol 8:771–778, 1984.
9. Kiviat NB, Wolner-Hanssen P, Eschenbach DA, Wasserheit JN, Paavonen JA, Bell TA, Critchlow CW, Stamm WE, Moore DE, Holmes KK: Endometrial histopathology in patients with culture-proved upper genital tract infection and laparoscopically diagnosed scute salpingitis. Am J Surg Pathol 14:167–175, 1990.
10. Kurman RJ, Mazyr MT: Benign diseases of the endometrium. In Kurman RJ (ed): Blaustein's Pathology of the Female Genital Tract. 4th ed. New York: Springer-Verlag, pp 367–409, 1994.
11. Payan H, Daino J, Kish M: Lymphoid follicles in endometrium. Obstet Gynecol 23:570–573, 1964.
12. Morris H, Edwards J, Tiltman A, Emms H: Endometrial lymphoid tissue: An immunohistological study. J Clin Pathol 38:644–652, 1985.
13. Kabawat SE, Mostoufi-Zadeh M, Driscoll SG, Bhan AK: Implantation site in normal pregnancy. A study with monoclonal antibodies. Am J Pathol 118:76–84, 1985.
14. Tabibzadeh SS, Bettica A, Gerber MA: Variable expression of Ia antigens in human endometrium and in chronic endometritis. Am J Clin Pathol 86:153–160, 1986.
15. Kamat BR, Isaacson PG: The immunocytochemical distribution of leukocytic subpopulations in human endometrium. Am J Pathol 127:66–73, 1987.
16. Bulmer JN, Lunny DP, Hagin SV: Immunohistochemical characterization of stromal leukocytes in nonpregnant human endometrium. Am J Reprod Immunol Microbiol 17:83–90, 1988.
17. Mizuuchi H, Kudo R, Tanura H, Kumai K, Sato K, Hashimoto M: Immunohistological identification of T and B cells in normal and malignant tissues of the uterine endometrium. Acta Obstet Gynaecol Jpn 41:349–356, 1989.
18. King A, Wellings V, Gardner L, Loke YW: Immunocytochemical characterization of the unusual large granular lymphocytes in human endometrium throughout the menstrual cycle. Hum Immunol 24:195–205, 1989.
19. Haller H, Radillo O, Rukavina D, Tedesco F, Candussi G, Petrovic O, Randic L: An immunohistochemical study of leukocytes in human endometrium, first and third trimester basal decidua. J Reprod Immunol 23:41–49, 1993.
20. Droegemueller W: Upper genital tract infections. In Herbst AL, Mishell DR Jr, Strenchever MA, Droegemueller W (eds): Comprehensive Gynecology. 2nd ed. St. Louis: Mosby-Year Book, pp 691–720, 1992.
21. Kim HM: Dysfunctional uterine bleeding. In Copeland LJ (ed): Textbook of Gynecology. Philadelphia: W.B. Saunders, pp 391–397, 1993.
22. Damjanov I: Infectious causes of infertility. In Damjanov I (ed): Pathology of Infertility. St. Louis: Mosby-Year Book, pp 105–143, 1993.
